# Phenotypic and molecular characterization of K64 putative hypervirulent carbapenem-resistant *Klebsiella pneumoniae*

**DOI:** 10.1371/journal.pone.0351561

**Published:** 2026-06-25

**Authors:** Chunhong Shao, Jian Bao, Xinying Wang, Yan Jin, Ren Ren, Wei Wang, Meijie Jiang

**Affiliations:** 1 Department of Clinical Laboratory, Shandong Provincial Hospital Affiliated to Shandong First Medical University, Jinan, China; 2 Department of Bone and Joint Diseases, Yantai City Traditional Chinese Medicine Hospital, Yantai, China; 3 Department of Clinical Laboratory, The Affiliated Taian City Central Hospital of Qingdao University, Tai’an, China; 4 Intensive Care Department, The Affiliated Taian City Central Hospital of Qingdao University, Tai’an, China; Cornell University, UNITED STATES OF AMERICA

## Abstract

**Objectives:**

Although K64-type carbapenem-resistant hypervirulent *Klebsiella pneumoniae* (CR-hvKp) has been reported, its molecular characteristics remain unclear. This study aimed to characterize the phenotypic and molecular features of K64 putative hypervirulent CR-*K. pneumoniae* (phvKp)..

**Methods:**

Two K64 putative hypervirulent CR-*K. pneumoniae* isolates were recovered from a patient in the emergency intensive care unit of a Chinese teaching hospital. Antimicrobial susceptibility testing was performed using broth microdilution method. Multilocus sequence typing (MLST) was used for molecular typing. Virulence was assessed using a mouse infection model, and whole-genome sequencing was performed to analyze resistance and virulence genes.

**Results:**

Both isolates were resistant to aztreonam, cephalosporins, carbapenems, quinolones, and aminoglycosides, but susceptible to trimethoprim/sulfamethoxazole, colistin, tigecycline, and ceftazidime/avibactam. MLST identified both isolates as ST11. The mouse infection model showed elevated virulence of two clinical isolates, which was significantly higher than that of the classical non-hypervirulent reference strain ATCC 13883. Genomic analysis identified 133 virulence- and pathogen-associated genes, including those involved in fimbriae synthesis, iron acquisition, and enterobactin production. Both isolates harbored the rmpA gene, but string tests were negative. Sequence alignment revealed >80% similarity among 13 rmpA-harboring IncHI1B plasmids.

**Conclusions:**

K64 ST11-type phvKp has emerged in China, exhibiting multidrug resistance and putative hypervirulence characteristics. The increase in virulence of *K.pneumoniae* involves multiple factors, and these factors act independently or the synergistic effect leads to elevated virulence potential.

## Introduction

*Klebsiella pneumoniae* (*K. pneumoniae*) is a prevalent nosocomial pathogen resulting in pneumonia, urinary tract infections, abdominal infections, and bacteremia. Over recent decades, it has become a key international concern because of increasing resistance and hypervirulence [1]. Historically, multidrug resistance and hypervirulence were deemed distinct phenotypes, with carbapenem-resistant *K. pneumoniae* (CRKp) strains exhibiting low virulence and hypervirulent *K. pneumoniae* (hvKp) strains remaining antibiotic-susceptible. However, carbapenem-resistant hypervirulent *K. pneumoniae* (CR-hvKp) has emerged worldwide, particularly in Asia [2].

CR-hvKp can evolve via horizontal transfer of carbapenem-resistance plasmids into hvKp or through CRKp acquiring hypervirulence plasmids. In particular, *K. pneumoniae* may produce CR-hvKp phenotypes by obtaining hybrid plasmids that carry resistance and virulence genes [3]. Despite this, knowledge of CR-hvKp remains limited. K1 and K2 serotypes are consistently related to hvKp [4]. Nonetheless, capsular-type K64 has been associated with hypervirulence [5]. Recently, several reports have been found on K64-type CR-hvKp [6–8], but the molecular features of the strains have not been illustrated. Herein, we distinguished the genotypic and phenotypic properties of two K64 ST11 putative hypervirulent carbapenem-resistant *K. pneumoniae* (phvKp) isolates obtained from a patient admitted to the emergency intensive care unit of a Chinese teaching Hospital.

## Materials and methods

### Bacterial strains

Two isolates of *K. pneumoniae* were obtained from a 61-year-old man with chronic obstructive pulmonary disease with complications of infection and pulmonary heart disease. The man had been hospitalized in 2019 in the emergency intensive care unit of Taian City Central Teaching Hospital, which is affiliated with Qingdao University in China. During hospitalization, endotracheal intubation and arterial catheterization were performed. Two isolates of *K. pneumoniae* were obtained from bronchoalveolar lavage fluid and blood cultures after hospitalization, which were designated as KP-2210B and KP-2210X. No history of traveling abroad was demonstrated. The isolates were retrospectively analyzed on 12/08/2024 after obtaining ethical approval (No. 2024-05-105). The data were anonymized/de-identified, and the authors had no access to information that could identify the individual patient.

After admission, the patient was exposed to piperacillin/tazobactam before blood culture was performed. The sensitivity of isolates to antibiotics was tested, and based on the results, the treatment regimen was changed to cefoperazone/sulbactam combined with tigecycline, but the patient’s condition continued to worsen. Ultimately, the patient’s family gave up treatment, and he was discharged five days after admission.

Herein, the methods received approval from the ethics committee of Taian City Central Hospital, affiliated with Qingdao University. The isolates were detected as *K. pneumoniae* via a Vitek-2 compact system and validated via a Vitek-MS system (BioMérieux, France). Phenotypic determination of carbapenemases was conducted via the carbapenem inactivation method (CIM) and an ethylenediaminetetraacetic acid (EDTA)-modified CIM (eCIM) examination.

### Serotype analysis and hypermucoviscosity phenotype

Using the string test, the hypermucoviscosity phenotype was assessed. Isolates were cultivated overnight on blood agar at 37 °C, and single colony streaking was conducted with a loop; a viscous string >5 mm indicated a positive result. Serotyping for K1, K2, K5, K20, K54, and K57 was conducted as previously mentioned [9].

### Antibiotic susceptibility assessment

Antimicrobial susceptibility was assessed via the VITEK-2 Compact system (BioMérieux, France). E-test (BioMérieux, France) was utilized to detect minimum inhibitory concentrations (MICs) of meropenem, imipenem, and ertapenem, while MICs of tigecycline and colistin were measured via broth microdilution (Bio-kont, China). Ceftazidime/avibactam susceptibility was evaluated via the Kirby–Bauer method (Oxide, USA). *E. coli* ATCC 25922 and *K. pneumoniae* ATCC 700603 were considered quality controls. Interpretations followed the 2023 EUCAST clinical breakpoints (www.eucast.org/clinical-breakpoint).

### Mouse lethality assessment

Pathogen-free C57BL/6 mice (males, 6–8 weeks old) were utilized to assess the virulence of the *K. pneumoniae* isolates (Changzhou Cavion Experimental Animal Co, Ltd., license no. SCXY (Su) 2011–0003). Ten mice were utilized per bacterial concentration, with *K. pneumoniae* ATCC 13883, a classical non-hypervirulent reference strain, as the control. An intraperitoneal injection of 0.1 mL of a 10⁵ CFU bacterial suspension in 0.9% NaCl was introduced into mice, and symptoms and death were monitored for 14 days. Mice were monitored every 12 hours for clinical signs of severe infection, including lethargy, ruffled fur, hunched posture, and difficulty breathing. Humane endpoints were strictly defined as a severe moribund state or the inability to voluntarily access food and water. Mice reaching these endpoints were euthanized immediately via CO_2_ inhalation followed by cervical dislocation to minimize suffering and distress. Survival rates of the mice were recorded daily for the entire 14-day observation period. All researchers involved in animal handling, monitoring, and euthanasia procedures had received standardized professional training in laboratory animal care, experimental operation, and humane euthanasia protocols. Ethics Statement. The retrospective analysis of clinical isolates received approval from the Ethics Committee of Taian City Central Hospital Affiliated to Qingdao University (Approval No. 2024-05-105), and individual informed consent was waived as per institutional requirements and national legislation. The mouse virulence assay was performed under the umbrella ethics approval of the project “Mechanistic study of hypervirulent *K. pneumoniae* Ent combined with LCN2 inducing ferroptosis in vascular endothelial cells leading to blood-brain barrier disruption” (Approval No. QDU-AEC-2024185, Ethics Committee of Medical College of Qingdao University, dated March 4, 2023), which covers the in vivo virulence evaluation of hypervirulent K. pneumoniae strains. All animal procedures were conducted in strict accordance with institutional guidelines for animal welfare.

### Multilocus sequence typing (MLST)

MLST of *K. pneumoniae* was conducted as per the MLST Pasteur (http://www.pasteur.fr/recherche/genopole/PF8/mlst/*K.pneumoniae*.html). Seven conserved housekeeping genes (*gapA*, *infB*, *phoE*, *mdh*, *pgi*, *rpoB*, and *tonB*) underwent amplification, sequencing, and comparing to those in the MLST databases.

### Genomic analysis

The draft genome of KP-2210X was generated by Shanghai Biozeron Biotechnology (Shanghai, China) using an Illumina HiSeq Xten platform. A TruSeq Nano DNA LT shotgun library was prepared for paired-end sequencing. Raw reads were processed with CASAVA v1.8.23 and assembled using SOAPdenovo. The annotation of Contigs >500 bp was conducted using the NCBI Prokaryotic Genome Annotation Pipeline (PGAP) and the “Rapid Annotation using Subsystem Technology” (RAST) (http://rast.nmpdr.org/). The annotation of genomic sequences was also conducted via the databases of the Kyoto Encyclopedia of Genes and Genomes (KEGG) (https://www.genome.jp/kegg/pathway.html) to assess the metabolic pathways. The Virulence Factor Database (VFDB) (http://www.mgc.ac.cn/VFs/main.htm) was utilized for the prediction of the bacterial virulence factors and pathogen-host interaction (PHI) factors.

### Phylogenetic tree construction and collinearity analysis

Plasmids carrying *rmpA* were retrieved using the query “*rmpA* and plasmid” from the NCBI database. Among 624 plasmids, 12 were detected as belonging to the IncHI1B incompatibility group. KP-2210X.p1 was compared with these 12 *rmpA*-harboring IncHI1B plasmids. Plasmid sequences were annotated for protein-coding genes using Prodigal (http://compbio.ornl.gov/prodigal/). A phylogenetic tree of the 13 plasmids was constructed using OrthoFinder’s STAG algorithm based on homologous protein clusters.

Collinearity analysis was conducted via Easyfig 2.2.5 software (http://mjsull.github.io/Easyfig/). The protein sequence was compared to the ISFinder database to obtain different types of mobile components, and then different colors of the components were displayed in a diagram based on their types.

## Results

### Resistance proﬁles

Table 1 displays the antimicrobial susceptibility profiles of the CRKp isolates. Both isolates exhibited resistance to ampicillin, ampicillin/sulbactam, aztreonam, and piperacillin/tazobactam, as well as all tested cephalosporins, quinolones, carbapenems, and aminoglycosides. However, they remained susceptible to colistin, tigecycline, trimethoprim/sulfamethoxazole, and ceftazidime/avibactam. mCIM tests were positive, whereas eCIM tests were negative. The isolates carried *bla*_KPC-2_, *bla*_SHV-11_, *bla*_TEM-1_, and *bla*_CTX-14_, accounting for β-lactam resistance; *rmtB*, conferring aminoglycoside resistance; and *qnrD*, potentially contributing to quinolone resistance.

**Table 1:** The antibiotic susceptibility of two K64 phvKp (μg/ml).

Antimicrobial Agents	MIC(mg/L)		
	KP2210B	KP2210X	Interpretation
KZ	>64	>64	R
CXM	>16	>16	R
CRO	>32	>32	R
CAZ	>16	>16	R
FEP	>64	>64	R
FOX	>16	>16	R
ATM	>16	>16	R
SAM	>16/8	>16/8	R
TZP	>64	>64	R
ETP	>32	>32	R
IPM	>32	>32	R
MEM	>32	>32	R
CN	>8	>8	R
AK	>32	>32	R
CIP	>2	>2	R
LEV	>4	>4	R
SXT	<=2/38	<=2/38	S
TGC	0.5	0.5	S
COL	0.5	0.5	S
CZA*	25	26	S

KZ:cefazolin; CXM:cefuroxime; CRO:ceftriaxone; CAZ, ceftazidime; FEP, cefepime; FOX,cefoxitin; ATM, aztreonam; SAM, ampicillin sulbactam; TZP, piperacillin/tazobactam; ETP, ertapenem; IPM, imipenem; MEM, meropenem; CN:gentamicin; AK:amikacin; CIP:ciprofloxacin; LEV:levoﬂoxacin; SXT: trimethoprim-sulfamethoxazole; TGC: tigecycline; CO: colisin; CZA: ceftazidime/avibatan;*:Diameter of antibacterial zone (mm); R, resistant; S, susceptible.

### MLST and virulence

**Fig 1:**
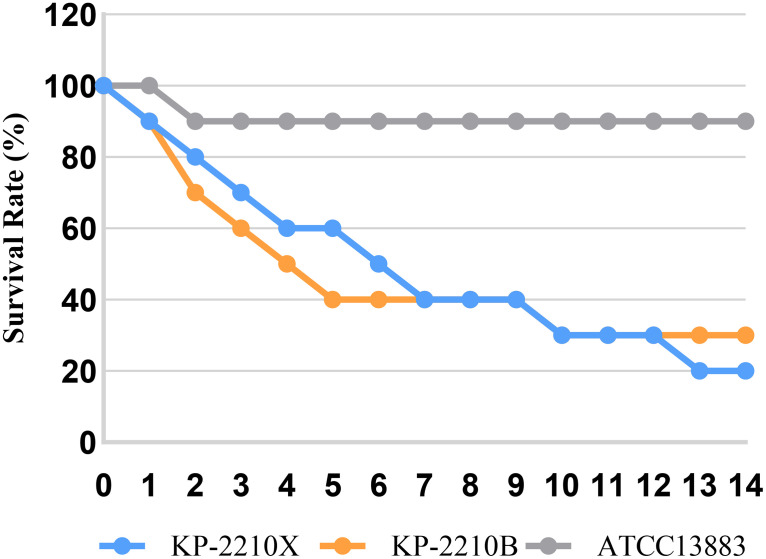
The survival rate of mouse infected by two phvKp isolates. *K. pneumoniae ATCC 13883* served as the control.

MLST analysis illustrated that both isolates were of the ST11 type, which is the most prevalent CRKp in China. Both isolates showed negative string-test results. Based on the polymerase chain reaction (PCR) amplification results, neither isolate is K1, K2, K5, K20, K54, or K57 serotypes. Furthermore, six virulence genes (*fimH, iucB, mrkD, rmpA, uge,* and *wabG*) were positive. In the mouse infection experiment, the virulence of the two isolates was similar, and their virulence was higher than that of ATCC 13883.The average survival rates of mice on days 5 and 10 were 50% and 30%, respectively. By day 14, mortality among mice infected with KP-2210X and KP-2210B was 80% and 70% respectively. Nonetheless, mortality among *K. pneumoniae* ATCC 13883 -infected mice was only 10% **(**Fig 1).

### Genomic characterization of KP-2210X and *rmpA*-harboring Plasmid

The draft genome of KP-2210X measured 5,455,575 bp with a GC content of 56.91%, and 5,422 genes were annotated. KEGG analysis identified 3,541 genes associated with 29 biological functions. KP-2210X was typed as serotype K64 and carried a few antibiotic resistance genes, consistent with its antibiotic susceptibility profile.

Sequencing showed that KP-2210X carried four plasmids: KP-2210X.p1, KP-2210X.p2, KP-2210X.p3, and KP-2210X. p4, which had sizes of 151,020, 78,504, 10,060, and 4,496 bp, respectively. Furthermore, 133 virulence-related genes were predicted using the VFDB database, which were related to fimbrial adherence, antiphagocytosis, endotoxin, immune evasion, iron uptake, the T6SS secretion system, and serum resistance. The virulence factor *rmpA* was positioned on the largest plasmid, KP-2210X.p1. A total of 170 antimicrobial resistance-related genes were also determined, among which *bla*_KPC−2_, *bla*_TEM−1_, and *rmtB* were located on KP-2210X.p2, while the rest were located on the genome of KP-2210X.

### Characterization of *rmpA*-harboring Plasmid

**Fig 2:**
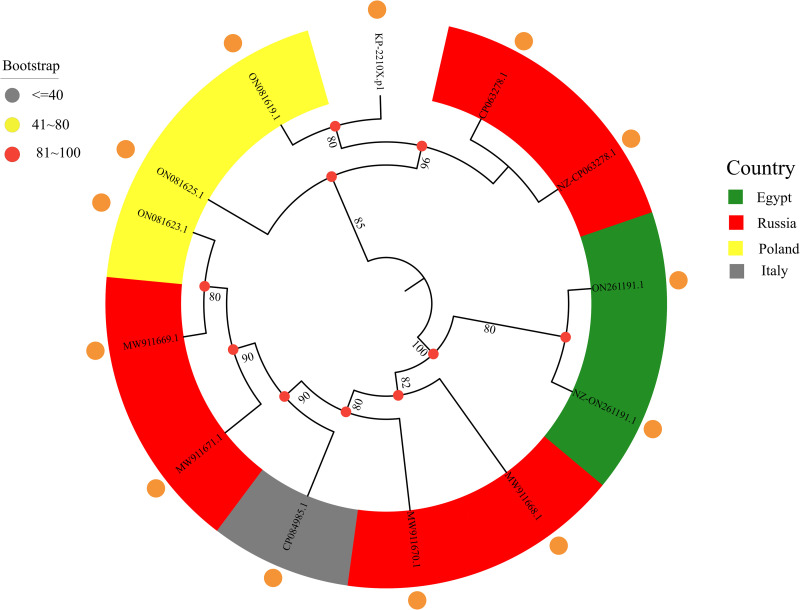
A phylogenetic tree of 13 IncHI1B plasmids harboring *rmpA.* Note: the numbers on the branch indicate the branch reliability, The closer the value is to 100, the higher the reliability is. The branch length represents the evolutionary distance, which is calculated by the average number of substitutions per nucleotide.

**Fig 3:**
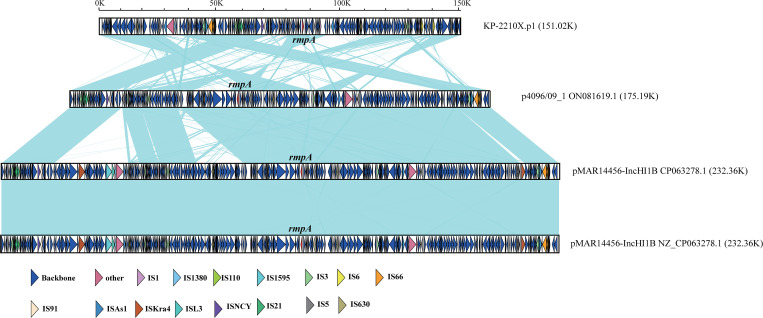
Linear comparison of KP-2210X.p1 with three IncHI1B plasmids harboring *rmpA* genes, mobile elements, and other features are colored based on function classification. Shading denotes regions of homology (>95% nucleotide identity).

KP-2210X.p1 is a 151,020-bp plasmid belonging to the IncHI1B incompatibility group. The full published sequences of 12 IncHI1B plasmids carrying *rmpA* were downloaded and compared to that of KP-2210X.p1 **(**Fig 2**)**. The isolation of all 12 other plasmids was conducted from *K. pneumoniae*, and they were submitted after 2021, and their source countries included Egypt, Russia, Poland, and Italy. Sequence alignment revealed that 13 plasmids exhibited remarkably stable phylogenetic relationships, with bootstrap values consistently exceeding 80%. KP-2210X.p1 showed the highest similarity to plasmids ON081619.1, CP063278.1, and NZ_CP063278.1. BLAST-based linear comparative genomics of these four plasmids demonstrated largely conserved genomic content. It can be observed that there are several insertions or substitutions of insertion sequences in KP-2210X.p1, such as IS91, which may be related to changes in biological traits such as bacterial morphology (Fig 3).

## Discussion

Over the past decade, CR-hvKp has attracted substantial attention. Herein, the recovery of the two phvKp isolates was conducted from bronchoalveolar lavage fluid and blood cultures of a 61-year-old man. The patient’s clinical course exhibited characteristic features of phvKp infection [10], including severe disease resulting from bloodstream invasion. Furthermore, the multidrug resistance of the bacteria poses great challenges to anti-infection treatment. The illness showed rapid progression and high severity in this patient.

According to MLST and genomic analysis, both isolates in the present study were of the ST11-K64 type, which is the most prevalent CRKp in China [11]. Previous study also showed that K64 isolates presented distinctive virulence-correlated characteristics, most of which belonged to ST11 [12]. Phylogenetic analysis indicates that ST11-K64 developed from an ST11-K47–like ancestor via recombination and has gradually substituted ST11-K47. ST11-K64 is related to significantly higher 30-day death and improved environmental survival [13]. Genomic analysis of KP-2210X identified 133 virulence-associated genes involved in capsule synthesis, flagellar motility, iron acquisition, immune evasion, and serum resistance. The mouse infection experiment also confirmed that the virulence of the two clinical isolates was significantly higher than that of *K. pneumoniae* ATCC 13883. However, our mouse virulence study used a single inoculum dose, lacked independent experimental repeats, and did not include a recognized hvKp positive control, which limits the statistical power and generalizability of the putative hypervirulence classification.

Up to now, *rmpA* and *magA* are the predominant factors directly linked to hvKp virulence [14]. *rmpA* and *rmpA2* encode transcriptional controllers that enhance capsule generation and may reside on the chromosome or on large virulence plasmids. *rmpA/rmpA2* show a strong correlation with the hypermucoviscous phenotype, and *rmpA* is a biomarker for identifying potential hvKp strains [15]. Herein, both hypermucoviscosity-negative isolates were nevertheless positive for *rmpA*. Based on earlier studies, the depolymerase contained in bacteriophages isolated from K64 *K. pneumoniae* can specifically degrade bacterial capsules, significantly improving bacterial sensitivity to serum and neutrophils [16]. To analyze the factors that affect *rmpA* activity, we analyzed the sequence characteristics of KP-2210X.p1, in which *rmpA* was located. This plasmid is 151 kb and belongs to the IncHI1B incompatibility group. It is possible that the insertion or translocation of introns in the surrounding environment of *rmpA* may result in abnormal expression and may be related to the phenotypic characteristics of KP-2210X.

In addition, hvKp also has several other microbiological characteristics associated with invasive infections, which are closely related to bacterial adhesion, anti-serum bactericidal activity, anti-phagocytosis, and cell colonization [17,18]. Acquiring iron is critical for bacterial survival and replication, and *K. pneumoniae* achieves this by secreting siderophores that tightly bind extracellular iron and transport it back into the cell [19]. Although siderophore content and expression vary among strains, efficient iron sequestration is a major contributor to pathogenicity [20]. The KP-2210X.p1 genome encodes 36 siderophore-associated genes, encompassing aerobactin, enterobactin, salmochelin, and yersiniabactin. In addition, types 1 and 3 fimbriae—key adhesion-related virulence factors—were identified and are encoded by the *fim* and *mrkABCD* gene clusters, respectively. In addition, KP-2210X also contains various other virulence factors, such as lipopolysaccharides, which can prevent the complement system from binding to bacteria and inhibit the complement pathway [21].

*bla*KPC-2 is the predominant carbapenemase in CRKp in China [22,23]. Similarly, herein, both phvKp isolates produce *bla*_KPC-2_. Besides *bla*_KPC-2_, both isolates harbored *bla*_TEM-1_, *bla*_SHV−11_, and *bla*_CTX−14_ genes. These resistance genes resulted in β-lactam resistance in both isolates. Both strains also have aminoglycoside and quinolone resistance, which limits the choice of antibiotics. Most existing treatment protocols for phvKp are similar to the treatment protocols for CRKp [24]. However, antibiotic selection and treatment regimens should be tailored to the strain’s phenotypic and genotypic profile, as well as the patient’s clinical status and underlying conditions.

## Conclusion

This investigation characterizes the phenotypic and molecular features of a K64-ST11 putative hypervirulent *K. pneumoniae (*phvKp) isolate that caused multisystem infection in an individual with chronic obstructive pulmonary disease and pulmonary heart disease. The K64 phvKp strain exhibits putative virulence-associated features that warrant further validation in multi-dose mouse infection models with appropriate hypervirulent and classical controls. However, the clinical therapeutic options are limited due to the multidrug resistance of this strain. Given its increasing prevalence, these findings underscore the potential of K64 phvKp to emerge as a significant public health threat.
